# G-8 indicates overall and quality-adjusted survival in older head and neck cancer patients treated with curative radiochemotherapy

**DOI:** 10.1186/s12885-015-1800-1

**Published:** 2015-11-09

**Authors:** Lies Pottel, Michelle Lycke, Tom Boterberg, Hans Pottel, Laurence Goethals, Fréderic Duprez, Sylvie Rottey, Yolande Lievens, Nele Van Den Noortgate, Kurt Geldhof, Véronique Buyse, Khalil Kargar-Samani, Véronique Ghekiere, Philip R. Debruyne

**Affiliations:** Kortrijk Cancer Centre, General Hospital Groeninge, campus loofstraat, Cancer Centre, Loofstraat 43, B-8500 Kortrijk, Belgium; Department of Radiation Oncology, Ghent University Hospital, De Pintelaan 185, B-9000 Ghent, Belgium; Department of Public Health and Primary Care, Subfaculty of Medicine, Catholic University Leuven Kulak, Etienne Sabbelaan 53, B-8500 Kortrijk, Belgium; Department of Medical Oncology, Ghent University Hospital, De Pintelaan 185, B-9000 Ghent, Belgium; Department of Geriatrics, Ghent University Hospital, De Pintelaan 185, B-9000 Ghent, Belgium; Department of Internal Medicine, Jan Yperman Hospital, Briekestraat 12, B-8900 Ypres, Belgium; Department of Internal Medicine, General Hospital OLV Lourdes, Vijfseweg 150, B-8790 Waregem, Belgium; Department of Oncology, Centre Hospitalier de Wallonie Picarde, RHMS, Chaussée de Saint-Amand 80, B-7500 Tournai, Belgium; Department of Geriatrics, General Hospital Groeninge, Reepkaai 4, B-8500 Kortrijk, Belgium; Ageing & Cancer Research Cluster, Centre for Positive Ageing, University of Greenwich, Avery Hill Rd., SE9 2UG Eltham London, UK

**Keywords:** Geriatric assessment, Head and neck cancer, Older patient, EuroQol-5 dimensions, Long-term quality of life, Curative treatment, Quality-adjusted survival

## Abstract

**Background:**

Evidence-based guidelines concerning the older head and neck cancer (HNCA) patient are lacking. Accurate patient selection for optimal care management is therefore challenging. We examined if geriatric assessment is indicative of long-term health-related quality of life (HRQOL) and overall survival in this unique population.

**Methods:**

All HNCA patients, aged ≥65 years, eligible for curative radio(chemo)therapy were evaluated with the Geriatric-8 (G-8) questionnaire and a comprehensive geriatric assessment (CGA). Euroqol-5 dimensions (EQ-5D) and survival were collected until 36 months post treatment start. Repeated measures ANOVA was applied to analyse HRQOL evolution in ‘fit’ and ‘vulnerable’ patients, defined by G-8. Kaplan-Meier curves and cox proportional hazard analysis were established for determination of the prognostic value of geriatric assessments. Quality-adjusted survival was calculated in both patient subgroups.

**Results:**

One hundred patients were recruited. Seventy-two percent of patients were considered vulnerable according to CGA (≥2 abnormal tests). Fit patients maintained a relatively acceptable long-term HRQOL, whilst vulnerable patients showed significantly lower median health states. The difference remained apparent at 36 months. Vulnerability, as classified by G-8 or CGA, came forward as independent predictor for lower EQ-5D index scores. After consideration of confounders, a significantly lower survival was observed in patients defined vulnerable according to G-8, compared to fit patients. A similar trend was seen based on CGA. Calculation of quality-adjusted survival showed significantly less remaining life months in perfect health in vulnerable patients, compared to fit ones.

**Conclusions:**

G-8 is indicative of quality-adjusted survival, and should be considered at time of treatment decisions for the older HNCA patient.

**Electronic supplementary material:**

The online version of this article (doi:10.1186/s12885-015-1800-1) contains supplementary material, which is available to authorized users.

## Background

Head and neck cancer (HNCA) ranks the sixth most common cancer worldwide, with more than half a million diagnoses each year [1, 2]. Globally, approximately half of HNCA patients are 65 years or older at diagnosis, and their numbers are even increasing [3]. Despite recent progress in the management of HNCA, benefits in overall survival have been associated with increased therapy-related morbidity persisting for up to 12 to 36 months after treatment, and adversely affecting patients’ health-related quality of life (HRQOL) [4–7].

Treatment decisions for the older HNCA population are challenging since evidence-based guidelines from large randomised-controlled trials (RCTs) are lacking due to exclusion of the older patient based on age or co-morbidities [3, 8–10]. Treatment delivered to older HNCA patients has been reported to comply with the institutions policy in less than 50 % of cases [11]. Although radiochemotherapy is considered as an intensive treatment in oncology patients, at present, there are only few tools available that integrate functional factors to adequately select eligible older patients. Since mid-1990 implementation of Comprehensive Geriatric Assessment (CGA) has been suggested by multiple international cancer networks, as a key treatment approach for all patients aged 70 years and older at time of diagnosis [12–15]. A CGA is described as a multidimensional test battery that screens for impairment in different age-related domains including co-morbidity, function, physical performance, cognition, nutrition, emotional status, polypharmacy, social support and living environment [16]. Several trials have reported the value of CGA in discovering geriatric problems, as well as to predict treatment tolerance, morbidity and mortality in mixed oncology settings based on their functional age. The time and resource requirements associated with performing CGA, however, have hindered implementation in daily practice [17]. Though, recently, the short Geriatric-8 (G-8) screening instrument was reported to have on its own the capacity to identify vulnerable patients and showed prognostic value for functional decline and overall survival in a mixed oncology population [18]. Our research team validated the G-8 for use in the HNCA population [19]. Moreover, we reported serial CGA to be indicative for HRQOL before and during treatment, and suggest its use to guide supportive care management during radiochemotherapy [20].

In light of the late toxicity often associated with radiochemotherapy [21, 22], the impact on daily functioning or thus patients’ HRQOL, might be more important to examine in an older HNCA population than the measurement of the classical hard end-points, such as disease-free or overall survival. Moreover, stratification of the older population into ‘fit’ and ‘vulnerable’ patients, based on geriatric assessments, could provide more reliable results about treatment tolerability and long-term outcome in these specific patient subgroups [23]. Previous studies have assessed long-term HRQOL in HNCA patients [24–28], however, to our knowledge, no study provided HRQOL based on subgroups distinguished by geriatric assessments for the older HNCA patient treated with curative intended radiochemotherapy.

This prospective trial was, therefore, designed to document the HRQOL, through the EuroQol-5 dimensions (EQ-5D) questionnaire, in both ‘fit’ and ‘vulnerable’ older HNCA patients from treatment start to long-term follow-up. Moreover, we also aimed to analyse the value of geriatric assessments to predict long-term HRQOL, overall survival, and quality-adjusted survival in both subgroups.

## Methods

### Study population

The trial involved older cancer patients, aged ≥65 years, with a histologically confirmed diagnosis of squamous cell carcinoma of the head and neck, eligible for curative primary or adjuvant radiotherapy, with or without concomitant systemic therapy. Tumours of the parotid gland or nasal cavity and paranasal sinuses were excluded. Other exclusion criteria were: distant metastases, another non-cured cancer, except for a squamous or basal cell carcinoma of the skin, and inability for adequate communication in Dutch or French. Patients were consecutively recruited from January 2010 until April 2012 upon presentation at the departments of radiation oncology at the General Hospital Groeninge (Kortrijk, Belgium) and the Ghent University Hospital (Ghent, Belgium) [19, 20]. The majority of patients were treated with intensity-modulated radiation therapy at a dose of 2.0 Gy per day, 5 days per week, for a total dose of 70 Gy in the primary, or 66 Gy in the adjuvant setting. Ten patients received conformal radiotherapy because of their poor general condition. Chemotherapy varied between weekly (40 mg/m^2^) or 3-weekly cisplatin (100 mg/m^2^) or weekly cetuximab (start dose of 400 mg/m^2^ one week prior to radiation therapy, followed by weekly 250 mg/m^2^) depending on their general condition and the treatment centre. Written informed consent was obtained from all included patients. The trial was approved by the Ethics Committees of the Ghent University Hospital (Ghent, Belgium) and the General Hospital Groeninge (Kortrijk, Belgium).

### Study design

An observational, multicentre, prospective study was performed. Patients were assessed with the Vulnerable Elders Survey-13 (VES-13) and the G-8 questionnaire, two screening tools [29, 30], and a full CGA as the gold standard, once before initiation of treatment (Week 0, W0), and again during the 4^th^ week (W4) of their treatment. In accordance with previous research, patients exhibiting no impairments or in only one domain within CGA were defined as ‘fit’ whilst patients exhibiting impairments in two or more domains within the CGA were defined as ‘vulnerable’ [19, 31–33]. The measures within the CGA tool are outlined in two prior publications performed by our research team [19, 20]. Since the initiation of our clinical trial in January 2010, the value of the VES-13 for use in the older population has been questioned [34, 35]. Moreover, it showed low sensitivity in the identification of vulnerable older HNCA patients [19]. For that reason, classification of patients by VES-13 was not included in the current analysis. The G-8, on the contrary, has been validated by several independent researchers in a mixed oncology population, and more specifically – by our own research team – in the target population [17, 19, 36]. Moreover, its prognostic value has been demonstrated in a mixed oncology population [18, 37]. Patients scoring 14 or less out of 17 on the G-8, were considered vulnerable.

**Fig. 1:**
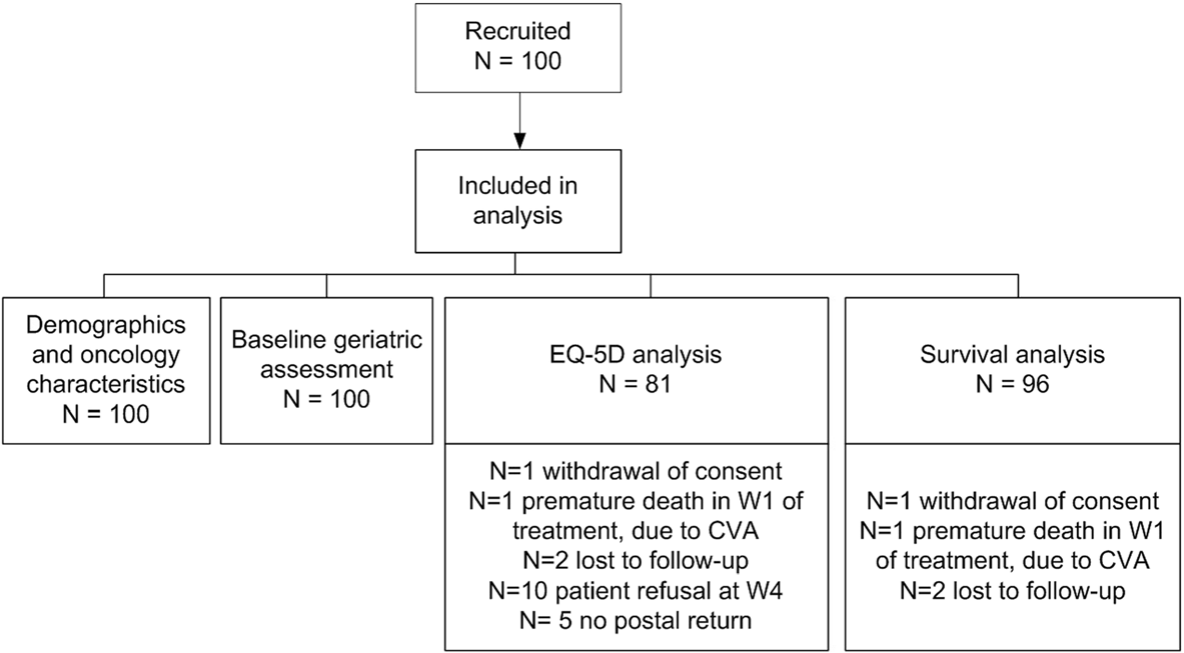
Patient flowchart. Abbreviations: W, week; CVA, cerebrovascular accident

At both time-points the EQ-5D questionnaire, as a measure of HRQOL, was self-completed or completed through patient interview when required. In addition, it was completed through a postal survey at 2, 5, 12, 24 and 36 months (M) after treatment start. A patient flowchart is presented in Fig. 1.

The EQ-5D is one of the most commonly used generic questionnaires to measure HRQOL, and has been developed by the EuroQol Group [38, 39]. It enables a self-reported description of the subjects’ current health in five dimensions i.e., mobility, self-care, usual activities, pain/discomfort and anxiety/depression. The subject is asked to grade their own current level of function in each dimension into one of three degrees of disability (severe, moderate or none). The combination of these with the conditions “death” and “unconscious” enables description of 245 different health states.

We applied the mathematical representation of the model developed by Cleemput, which was based on 25 health states that were obtained by 548 Flemish (Belgian) respondents included in the final dataset [40]. Based on this model, for each health state an utility score can be deducted, called the EQ-5D index score, which represents the patients’ description of their own health and how this health state relates to the health state of the general population [38, 41]. A score of 1 indicates perfect health, 0 indicates death. Negative scores indicate ‘worse than death’, and represent health states where patients experience at least severe disability in three dimensions, in combination with moderate or severe disability in the remaining dimensions.

Baseline demographic data, clinical characteristics, details of their medical history, therapy regimen, and overall survival were collected through the medical records.

### Statistical analysis

Demographic, oncological, geriatric, HRQOL and survival data were analysed descriptively. Classification of patients as ‘fit’ or ‘vulnerable’ was based on baseline (W0) CGA or G-8 assessments. Repeated measures ANOVA were applied to evaluate the difference in HRQOL evolution, measured with the EQ-5D questionnaire, between both ‘fit’ and ‘vulnerable’ patients, and corrected for confounders such as age, gender, civil state, living situation, tumour diagnosis, tumour stage, and applied treatment. Spearman correlations were performed to indicate potential correlations between EQ-5D index scores, G-8, and the number of abnormal tests within CGA. Kaplan-Meier curves were established to analyse the prognostic value of patient classification based on G-8 and CGA. A cox proportional hazard analysis was performed to confirm the prognostic value of the different geriatric assessments, after correction for confounders such as age, gender, civil state, tumour diagnosis, tumour stage, and applied treatment. Variables were selected by backward elimination. Quality-adjusted survival within 36 months of follow-up was calculated for evaluation of the HRQOL in the remaining life months, and Mann–Whitney *U* test was applied for comparison between both subgroups. Quality-adjusted life months were calculated through resolution of the following integral: *QALY = ∑ ∫ P*_*s*_*(t)Q*_*s*_*(t)dt* where *P*_*s*_*(t)* represents the probability of survival, and *Q*_*s*_*(t)* represents the HRQOL [42], measured with the EQ-5D index score, at the seven different evaluation time points.

All analyses were performed using Prism® software (GraphPad Prism 5, Inc., La Jolla, CA) and SPSS or SAS software (version 20; IBM SPSS Statistics, Chicago, IL; version 9.3; SAS Institute Inc., Cary, NC, USA). Statistical significance was assumed when *P* < 0.05. Since this manuscript comprises (prespecified) secondary endpoints, all *P*-values should be considered as explorative, and thus have a merely hypothesis-generating value.

## Results

### Study population characteristics

One hundred patients were recruited. An overview of demographic and oncological characteristics is presented in Table 1.

**Table 1:** Demographic and oncological characteristics of the study population

	Study population	Fit patients^a^	Vulnerable patients^a^
	*N* = 100	*N* = 32	*N* = 68
Demographic characteristics		Median (range)	
Age	72.0 (65.0– 86.0)	72.0 (65.0–84.0)	72.0 (65.0–86.0)
		%	
Gender			
Male	86	28	58
Female	14	4	10
Social status			
Married/living together	72	27	45
Profession			
Labourer	44	12	32
Employee	31	8	23
Self-employed	25	12	13
Clinical characteristics			
Tumour location			
Oral cavity	15	3	12
Oropharynx	22	6	16
Hypopharynx	10	0	10
Nasopharynx	2	0	2
Supraglottis	18	4	14
Glottis	24	14	10
Subglottis	3	1	2
Occult primary	6	4	2
Tumour stage			
Early stage (I-II)	31	15	16
Late stage (III-IVb)	69	17	52
Therapy initiation			
Primary radiotherapy			
Alone	40	14	26
With chemotherapy (cisplatin)	21	8	13
With biotherapy (cetuximab)	8	1	7
Adjuvant radiotherapy			
Alone	21	7	14
With chemotherapy (cisplatin)	8	2	6
With biotherapy (cetuximab)	2	0	2

^a^classification according to G-8, assessed at W0

Seventy-two percent of patients were considered vulnerable, thereby presenting with at least two abnormal test results within the full CGA, assessed at W0. Patients presented with deficiencies in two (29 %), three (14 %), four (16 %), five (7 %) and all but one (6 %) of the domains within CGA. Of the 28 % of patients who were defined fit, 8 % did not show a single deficiency on one of the standardised tests within CGA. The majority of patients presented with severe-grade comorbidities (CIRS-G, 77 %), while half of patients showed difficulties in community functioning (IADL, 52 %) and nutritional parameters (MNA, 48 %). Approximately one third of patients showed problems with gait and balance (Tinetti, 29 %), and a quarter of patients did not succeed in self-care (ADL, 16 %), or showed signs of cognitive impairment (MMSE, 16 %) and depression (GDS, 17 %) [19, 20].

According to the G-8 (cut-off ≤14), 68 % of patients were considered vulnerable. Patients showed a median G-8 score of 13.0 (Q1,Q3: 10.0, 15.0), with a minimum and maximum score of respectively 3.0 and 17.0. An overview of W0 and W4 CGA and G-8 data is presented in Table 2.

**Table 2:** Vulnerability percentage of HNCA patients, assessed by CGA or G-8, at W0 and W4

	Vulnerable patients at W0	Vulnerable patients at W4
	(*n* = 100; %)	(*n* = 87; %)
Geriatric-8 (G-8)	68.0	92.0
Comprehensive geriatric assessment (CGA)	72.0	83.9
Domains within CGA		
Activities of Daily living (ADL)	16.0	18.4
Instrumental activities of daily living (IADL)	52.0	70.1
Mini nutritional assessment (MNA)	48.0	75.9
Mini mental state examination (MMSE)	16.0	17.4
Geratric depression scale (GDS)	17.0	28.7
Tinetti gait and balance (Tinetti)	29.0	34.5
Cumulative illness rating scale for geriatrics (CIRS-G)	77.0	78.2

*W* week

Adapted from Pottel et al. [20]

### Short- and long-term health-related quality of life

EQ-5D data, as a measure of HRQOL, was complete for eighty-one patients. Nineteen patients had one or several missing assessments due to several reasons that are reported in Fig. 1. Post-treatment EQ-5D postal response was 90 %.

In general, a median EQ-5D index score of 0.66 (0.55, 0.76) was found for all patients prior to treatment start. A deterioration of HRQOL, defined by EQ-5D, towards a median score of 0.42 (0.26, 0.73) was reported during mid-therapy (W4). Median scores increased again towards baseline values 0.66 (0.29, 0.76) at end of treatment (2 M). During follow-up evaluations, patients reached median EQ-5D index scores of 0.66 (0.27, 0.76) at 5 M, however, values slowly deteriorated thereafter due to the increasing number of deaths (12 M: 0.64 (0.0, 0.76), 24 M: 0.29 (0.0, 0.76), and 36 M: 0.0 (0.0, 0.67)).

**Fig. 2:**
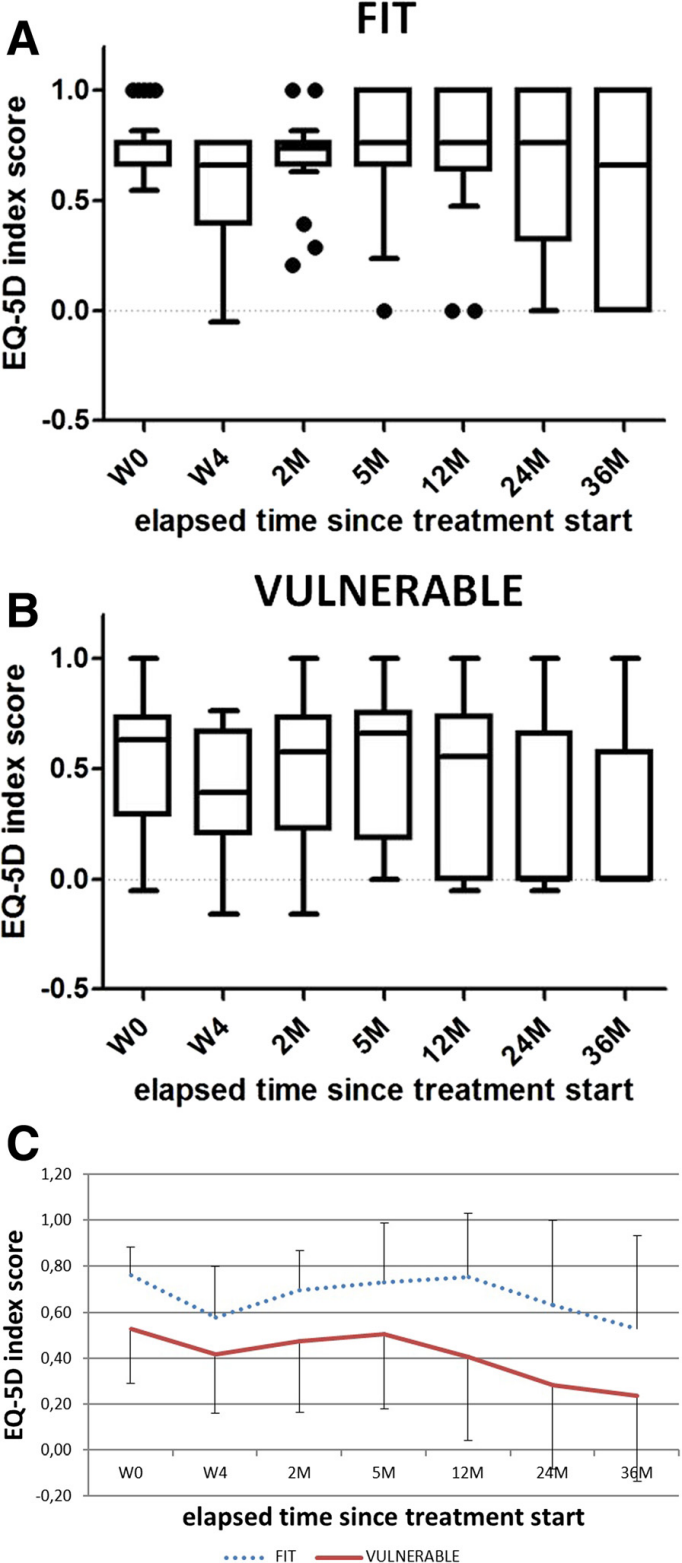
Evolution of health-related quality of life, assessed by EQ-5D, from treatment start to 36 months of follow-up. **a**-**b** Data presented as boxplots, graphically displaying median, inter-quartile range and minimum and maximum data values. **c** Data presented as mean ± standard deviation. **a** Evolution of HRQOL, assessed by EQ-5D, in ‘fit’ older HNCA patients, as defined by G-8. **b** Evolution of HRQOL, assessed by EQ-5D, in ‘vulnerable’ older HNCA patients, as defined by G-8. **c** Evolution of HRQOL, assessed by EQ-5D, in ‘fit’ and ‘vulnerable’ older patients

EQ-5D index scores for fit and vulnerable patients, as classified by G-8, are presented in Table 3 and Fig. 2. While fit patients regained baseline values at end of treatment, vulnerable patients, on the contrary, showed significantly lower EQ-5D index scores compared to fit patients, as well before, during and after treatment start (*p* < 0.05). Classification by CGA showed comparable results (data not shown).

**Table 3:** Short- and long-term health-related quality of life, represented by EQ-5D index score

	W0	W4	2 M	5 M	12 M	24 M	36 M
	EQ-5D index score [median (Q1, Q3)]						
General	0.66 (0.55, 0.76)	0.42 (0.26, 0.73)	0.66 (0.29, 0.76)	0.66 (0.27, 0.76)	0.64 (0.0, 0.76)	0.29 (0.0, 0.76)	0.00 (0.00, 0.67)
Fit^a^	0.76 (0.66, 0.76)	0.66 (0.39, 0.76)	0.74 (0.66, 0.76)	0.76 (0.66, 1.00)	0.76 (0.64, 1.00)	0.76 (0.32, 1.00)	0.66 (0.00, 1.00)
Vulnerable^a^	0.63 (0.29, 0.73)	0.39 (0.21, 0.67)	0.58 (0.23, 0.73)	0.66 (0.19, 0.76)	0.57 (0.00, 0.74)	0.00 (0.00, 0.66)	0.00 (0.00, 0.58)
	Spearman correlations [r_s_]^b^						
G-8	0.64	0.42	0.41	0.42	0.52	0.45	0.38
Abnormal CGA domains	−0.74	−0.46	−0.43	−0.45	−0.46	−0.34	−0.41

*W* week, *M* month, *G-8* geriatric-8, *EQ-5D* euroqol-5 dimensions

^a^classification based on G-8 assessed at W0, ^b^spearman correlations between EQ-5D index score and respectively G-8 and the number of abnormal CGA domains

As presented in Table 3, Spearman correlations showed a statistically significant positive correlation between patients’ G-8 score and the EQ-5D index score at different time-points before, during and after treatment (*P* < 0.001). In accordance, a statistically significant negative correlation was found between the number of abnormal CGA domains and the corresponding EQ-5D index score (*P* < 0.001).

Repeated measures ANOVA revealed a statistically significant time-effect of EQ-5D index scores (*P* < 0.0001), a significant effect of vulnerability as classified by G-8 or the number of positive CGA domains at treatment start (*P* < 0.0001), and a significant effect of advanced-stage cancer (*P* < 0.001) as independent predictors of low HRQOL values (Additional file 1: Table S1).

### Prognostic value of geriatric assessments

Survival data were missing for four patients (Fig. 1). Seven patients died during treatment or in the first week after end of treatment. During follow-up, 10.4 %, 25.0 %, 40.6 % and 51.0 % of the total study population had died at respectively 5, 12, 24 and 36 M. Median(Q1,Q3) survival was respectively 1095 days (1018, 1095) and 687 days (338, 1095) days for fit and vulnerable patients, assessed with G-8.

**Fig. 3:**
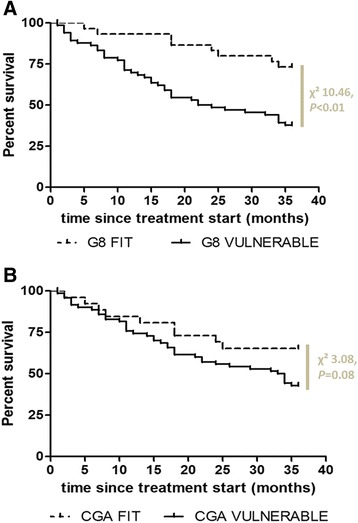
Overall survival, represented by Kaplan-Meier curves, of the ‘fit’ and ‘vulnerable’ older HNCA patient. **a** Overall survival of patients classified as ‘fit’ or ‘vulnerable’, by G-8. **b** Overall survival of patients classified as ‘fit’ or ‘vulnerable’, by CGA. Log-rank test was applied to measure the difference between the curves

Kaplan-Meier curves revealed a statistically significant lower overall survival in patients defined vulnerable according to G-8 (log-rank *χ*^2^ = 10.46, *P* < 0.01), compared to fit patients. Likewise, a trend towards statistical significant lower survival in vulnerable patients, compared to fit patients was seen when defined by CGA (*χ*^2^ = 3.08, *p* = 0.08) (Fig. 3).

Cox proportional hazard analysis indicated advanced-stage cancer (*P* < 0.05) and vulnerability according to G-8 (*P* < 0.01) as independent predictors of mortality after curative radio(chemo)therapy. Within the model for CGA, advanced-stage cancer (*P* < 0.001) and male gender (*P* < 0.05) were predictive for mortality, but not CGA (*P* = 0.12) (Additional file 2: Table S2).

### Quality-adjusted survival

Calculation of the quality-adjusted survival within 36 M of follow-up showed significantly lower remaining life months in perfect health in the vulnerable (V) group, compared to the fit (F) group of patients, as classified by the G-8 (F: 23.3 (18.2, 27.4), V: 8.8 (2.8, 15.0), *p* < 0.0001) and full CGA (F: 21.8 (11.2, 25.4), V: 9.9 (3.8, 15.9), *p* < 0.001).

## Discussion

Lack of evidence-based guidelines concerning appropriate care for the older HNCA patient challenges treating physicians in the trade-off between quality of life and survival [3, 11]. Although implementation of geriatric assessments has been proposed by several international cancer networks in latest years [13, 15, 43], we were the first to examine the feasibility of performing serial CGA during radiochemotherapy in this target population [19, 20]. In the current final manuscript on these data, we demonstrate - for the first time - that vulnerable patients, as classified by G-8 or CGA, show significantly lower HRQOL within their remaining life months compared to fit patients. Moreover, our results are suggestive for G-8 as an independent parameter for quality-adjusted survival.

At treatment initiation, approximately three quarter of patients were defined vulnerable according to CGA. A relatively low median EQ-5D index score of 0.66 was observed at presentation. This could partly be explained by the symptomatic, advanced-stage of the disease. Based on CGA, and without taking potential confounders into consideration, the proportion of fit or vulnerable patients was comparable in the early and advanced-stage diseased groups. However, patients with an advanced-stage cancer did show a significantly lower G-8 score than early stage patients (*P* < 0.01, data not shown). Since three of the eight questions in the G-8 questionnaire focus on nutritional problems, and advanced-stage HNCA is often associated with malnutrition due to difficulty of deglutition or mastication, G-8 might slightly overestimate the proportion of vulnerable patients in case of an advanced-stage cancer. However, in the multivariate survival and EQ-5D analysis the effect of G-8 remained significant, after correction for confounders such as tumour stage.

In comparison, EQ-5D index scores ranging from 0.11 to 0.71 were described in literature for respectively older institutionalised patients with dementia [44], to older patients receiving post-acute rehabilitation in an outpatient [45] or day rehabilitation facility [46] setting. A significant deterioration of health state was seen at mid-treatment. Indeed, treatment is known to induce additional disability [47, 48]. Moreover, prior work by our research team showed a significant increase in the number of vulnerabilities, as measured by CGA, at mid-treatment. Also, we found that CGA is indicative of short-term HRQOL (during treatment). Indeed, all patients defined as ‘vulnerable’ showed significantly lower functional status, and higher symptom scores, as measured by EORTC QLQ C30 and HN35 questionnaire, compared to their ‘fit’ counterparts both prior to, and during treatment [20].

Although it is known that chronic radiation-induced toxicities are common in this population, no literature could be found regarding its impact on long-term HRQOL in the older HNCA patient. To the best of our knowledge, we are the first to provide data concerning long-term HRQOL and overall survival in subgroups of older HNCA patients, as classified by geriatric assessments.

Our data demonstrate that fit patients obtained HRQOL values comparable to baseline values already at treatment end, while vulnerable patients only regained their original health state at 5 months post treatment start. Moreover, fit patients were able to maintain a median HRQOL index of 0.76 until 24 months. In vulnerable patients, on the contrary, a fast decline in HRQOL was observed at one year onwards. Spearman correlations confirmed that a higher vulnerability score, based on CGA or G-8, was associated with a lower EQ-5D index score at all evaluated time points. In comparison, Ramaekers et al. reported an average EQ-5D index of 0.85 at 6 months post radio(chemo)therapy in 396 HNCA patients of all ages with no evidence of recurrent disease [28]. Although HRQOL is a very subjective matter, Kvamme et al. reported that cut-off points around 0.65–0.70 for the EQ-5D questionnaire indicate an acceptable health state across diseases [49]. According to this definition, the overall long-term HRQOL scores reported by vulnerable patients could thus be considered unacceptable. Since an EQ-5D index of “0” was assigned to patients who had died, the median health index represents an accurate reflection of the total population, unbiased by survivorship effects. Indeed, median EQ-5D index scores of the survivors range between 0.69–0.75 from 5 M post treatment initiation onwards. Surviving vulnerable patients show a significant lower EQ-5D index score at follow-up than their fit counterparts (data not shown). Kaplan-Meier analyses confirmed the significantly lower survival rate in patients defined vulnerable based on G-8. Although only a trend was seen between vulnerability according to CGA and lower survival, this could be explained by the cut-off of ≥2 positive tests within CGA that was used. There is at present no consensus on CGA content or the associated cut-off scores to categorise HNCA patients as fit, vulnerable or frail. Patients alive at 36 M did show a significantly lower number of positive CGA tests at treatment start, as compared to deceased patients (*p* < 0.01, data not shown). At 36 M, 64 % of vulnerable patients had died, in comparison to 30 % of fit patients. This is in agreement with prior publications reporting a prognostic value for the G-8 tool in a mixed oncology population [18, 37]. Moreover, the high mortality rate corresponds with a 5-year-overall survival rate of 30 % in advanced-stage HNCA patients of all ages, treated with curative intent [50, 51].

In agreement with the parameters reported by Sanabria et al. in an older HNCA population, we also found male gender and advanced clinical stage as independent predictors of mortality [52]. However, contrary to this publication, comorbidity was not retained in our cox proportional hazard model. This could be partly explained by the different use of comorbidity assessment tools. High CIRS-G scores were seen in the majority of HNCA patients in our study, due to cardiovascular and respiratory problems associated with tobacco and alcohol abuse. Moreover, Extermann described functional status to be independent of comorbidity in an older cancer population [53]. Also, at present, evidence for the prognostic value of comorbidity has been considered inconsistent [54].

In addition to the significant survival difference observed between both subgroups, quality-adjusted survival revealed that vulnerable patients – although treated with curative intent – have less than 10 remaining life months in perfect health, compared to approximately 24 months for fit patients. These data clearly state that the length of survival has to be weighed against the quality of survival. Hence, HRQOL and factors that could influence HRQOL should be considered important treatment outcomes when examining new treatment regimens in the older HNCA population. Also, patients’ subjective assessment should always have a central role upon any consideration of treatment.

The results of this trial should, however, be interpreted with caution, due to some study limitations. First, there is at present no consensus on CGA content or its definition of ‘vulnerability’ [17]. The G-8 tool, however, has in the meantime been validated in both a French mixed oncology population and the target population in specific [19, 30]. Moreover, it has been reported to have on its own the capacity to identify vulnerable patients and showed prognostic value for functional decline and overall survival in a mixed oncology population [18]. Therefore, we mainly focused on classification by G-8.

Second, because our objective was to measure the impact of radio(chemo)therapy on HRQOL, baseline geriatric assessments were performed prior to radiation treatment start, irrespective of a primary or adjuvant treatment setting. All patients in an adjuvant setting underwent surgery within approximately four weeks of the geriatric assessment, which could have had a possible impact on their general condition. However, no significant difference in the proportion of vulnerability, based on G-8 or CGA, could be found between the primary and adjuvant treatment subgroup (data not shown). Third, our population was a relatively young elderly population, with a median age of 72 years old. Although NCCN guidelines indicate 70 years of age as a clinically relevant breakpoint, we included patients aged 65 or older at enrolment [15]. This age was based on historical categorisation, as well as the knowledge that HNCA patients often present with a geriatric profile already before the age of 70, due to their often significant co-morbidities. Fourth, although the EORTC HRQOL questionnaires have been validated internationally and offer detailed information about both the functional and symptomatic health status of HNCA patients, we chose the EQ-5D questionnaire as a postal survey for long-term follow-up [55–57]. A potential limitation of the calculated EQ-5D index is that the value set on which the calculations are based, might not be representative for the older HNCA population [58]. Indeed, many HRQOL instruments have not been tailored to the special requirements of the older patient [59]. The study population with a median age of 72 years old, comprising both Flemish and Walloon patients, could have different views on quality of life and death compared to the healthy Flemish middle-aged population used as a value set. However, the tool has been reported to have good responsiveness [60] and was suggested by Rogers et al. for use in the HNCA population [27]. Moreover, since the EQ-5D only comprises five short questions, we considered it more suitable since it limits patient burden and in that way also encourages response rates. Indeed, with a response rate of 90 %, the numbers are significantly higher than the 64 % that is considered an acceptable response rate [27].

Fifth, the heterogeneity of treatment is common practice in this population [3]. Each individual’s treatment plan was discussed at the multidisciplinary oncology consult, according to local hospital guidelines and the experience of the treating physicians, who may or may not have been partly influenced by the geriatric assessment. In our study population, only a minority of early- and advanced-stage cancers were treated in an adjuvant setting (respectively 12.9 % vs 39.1 %). According to Sanabria et al., who reported that substandard treatment offered to oral and oropharyngeal cancer patients led to lower overall and cancer-specific survival [61], the lower quality-adjusted survival observed in vulnerable patients could also be related to an increased cancer-specific death. However, no significant difference in disease-related deaths (i.e. local recurrences, distant metastases, or treatment-related chronic toxicity), nor in applied chemotherapy or radiation therapy dose could be found between both fit and vulnerable patients (data not shown).

In addition, although our data underscore the value of geriatric assessments to indicate long-term HRQOL, the heterogeneity of applied treatment regimens impedes us from providing prospectively validated recommendations about integration of CGA in optimal treatment planning [62]. Incorporation of long-term HRQOL in a RCT setting, as is currently being performed by Paillaud et al. [63], could provide a cost-benefit measurement of CGA intervention in the target population, through calculation of quality-adjusted life years gained. Also, future phase II or III oncology trials should take long-term HRQOL in different subgroups of the older patient into account, to enable valid judgment regarding individual treatment tolerance and cost-benefits for the healthcare institution [23]. Finally, since HPV-status was unavailable for most patients at time of treatment, it was not incorporated in the analysis as a confounding factor [64]. Also, no data was collected regarding late radiation-induced toxicities.

## Conclusions

In conclusion, our data demonstrate that classification of patients as fit or vulnerable, based on G-8, is indicative of overall and quality-adjusted survival, and should be integrated at time of treatment decision for the older HNCA patient. Moreover, future clinical trials should integrate long-term HRQOL as a surrogate end-point in order to balance the trade-offs between late treatment toxicities and expected survival in stratified groups of the older HNCA population, as classified by geriatric assessments.

## Additional files

Additional file 1: Table S1. Repeated Measures ANOVA model of EQ-5D. (a) patient classification based on G-8. (b) patient classification based on CGA. (DOCX 20 kb) Additional file 2: Table S2. Cox proportional hazard model of overall survival. (a) prognostic value of G-8. (b) Prognostic value of CGA. Variables were selected by backward elimination. (DOCX 20 kb)
