# Supplementary material for: G-8 indicates overall and quality-adjusted survival in older head and neck cancer patients treated with curative radiochemotherapy
Source: BMC Cancer. 2015 Nov 9;15:875. doi: 10.1186/s12885-015-1800-1 (PMC4640221; doi:10.1186/s12885-015-1800-1)
Supplement: Additional file 1: Table S1. — Repeated Measures ANOVA model of EQ-5D. (a) patient classification based on G-8. (b) patient classification based on CGA. (DOCX 20 kb) [file 12885_2015_1800_MOESM1_ESM.docx]

**Additional file 1: Table S1**

**(A)**

| **Effect** | **DF** | **F-value** | **P-value** |
| --- | --- | --- | --- |
| **Age** | 1 | 0.87 | 0.35 |
| **Visit** | 1 | 24.76 | **<0.0001** |
| **Gender** | 1 | 1.23 | 0.27 |
| **Civil state** | 3 | 2.30 | 0.08 |
| **Living alone** | 2 | 0.94 | 0.39 |
| **Stage** | 3 | 5.05 | **0.002** |
| **CIRS-G** | 1 | 0.34 | 0.56 |
| **G-8** | 1 | 19.27 | **<0.0001** |

*Abbreviations: DF, degree of freedom; CIRS-G, cumulative illness rating scale for geriatrics; G-8, geriatric-8*

**(B)**

| **Effect** | **DF** | **F-value** | **P-value** |
| --- | --- | --- | --- |
| **Age** | 1 | 0.24 | 0.63 |
| **Visit** | 1 | 25.01 | **<0.0001** |
| **Gender** | 1 | 1.78 | 0.18 |
| **Civil state** | 3 | 0.86 | 0.46 |
| **Living alone** | 2 | 0.98 | 0.37 |
| **Stage** | 3 | 6.69 | **0.0002** |
| **Number of positive CGA domains** | 6 | 10.25 | **<0.0001** |

*Abbreviations: DF, degree of freedom; CGA, comprehensive geriatric assessment*
