# Supplementary material for: G-8 indicates overall and quality-adjusted survival in older head and neck cancer patients treated with curative radiochemotherapy
Source: BMC Cancer. 2015 Nov 9;15:875. doi: 10.1186/s12885-015-1800-1 (PMC4640221; doi:10.1186/s12885-015-1800-1)
Supplement: Additional file 2: Table S2. — Cox proportional hazard model of overall survival. (a) prognostic value of G-8. (b) Prognostic value of CGA. Variables were selected by backward elimination. (DOCX 20 kb) [file 12885_2015_1800_MOESM2_ESM.docx]

**Additional file 2: Table S2**

**(A)**

| **parameter** | **Parameter estimate** | **SE** | **Chi-square** | **p-value** | **Hazard ratio** | **95% hazard ratio confidence limits** | |
| --- | --- | --- | --- | --- | --- | --- | --- |
| **Stage IV** | 1.01 | 0.45 | 5.01 | **0.03** | 2.74 | 1.13 | 6.60 |
| **Stage III** | 0.23 | 0.53 | 0.20 | 0.66 | 1.26 | 0.45 | 3.55 |
| **Stage II** | -0.48 | 0.71 | 0.45 | 0.50 | 0.32 | 0.16 | 2.49 |
| **Vulnerability according to G-8** | 1.16 | 0.39 | 8.79 | **0.007** | 3.19 | 1.48 | 6.87 |

*Abbreviations: G-8, geriatric-8; SE, standard error*

**(B)**

| **parameter** | **Parameter estimate** | **SE** | **Chi-square** | **p-value** | **Hazard ratio** | **95% hazard ratio confidence limits** | |
| --- | --- | --- | --- | --- | --- | --- | --- |
| **Male gender** | 1.06 | 0.46 | 5.42 | **0.02** | 2.90 | 1.18 | 7.08 |
| **Stage IV** | 1.27 | 0.45 | 7.85 | **0.005** | 3.57 | 1.47 | 8.68 |
| **Stage III** | 0.26 | 0.53 | 0.25 | 0.62 | 1.30 | 0.46 | 3.66 |
| **Stage II** | -0.54 | 0.71 | 0.58 | 0.45 | 0.59 | 0.15 | 2.34 |
| **Vulnerability according to CGA** | 0.58 | 0.38 | 2.37 | 0.12 | 1.79 | 0.85 | 3.74 |

*Abbreviations: CGA, comprehensive geriatric assessment; SE, standard error*
